# The mediating role of general self-efficacy in the association between perceived social support and oral health-related quality of life after initial periodontal therapy

**DOI:** 10.1186/s12903-016-0227-7

**Published:** 2016-06-07

**Authors:** Lei Miao, Jingwen Feng, Liuzhong Wu, Shuwei Zhang, Ziming Ge, Yaping Pan

**Affiliations:** Department of Periodontics and Oral Biology, School of Stomatology, China Medical University, No. 117 Nanjing North Street, Heping District, Shenyang, Liaoning 110002 People’s Republic of China

**Keywords:** Oral health-related quality of life, Perceived social support, General self-efficacy, Periodontitis, Initial periodontal therapy

## Abstract

**Background:**

Although initial periodontal therapy can ease some physical and psychological discomforts from periodontitis and improve patients’ oral health-related quality of life (OHRQoL), it is also vital to find positive resources from psychological and social aspects to promote the overall OHRQoL. This study aims to explore the associations of perceived social support (PSS) and general self-efficacy with OHRQoL and the mediating role of general self-efficacy in PSS-OHRQoL association after initial periodontal therapy.

**Methods:**

A prospective case series study was conducted among consecutive outpatients with chronic periodontitis during the period of July 2014–April 2015. A total of 145 eligible patients responded to OHRQoL questionnaire and periodontal examination at baseline. About 4 to 5 weeks after initial periodontal therapy, 120 patients completed the second OHRQoL measurement and periodontal examination, along with PSS and general self-efficacy measurement. The Wilcoxon matched-pairs signed-rank test was used to determine the difference between baseline and post-treatment OHRQoL scores and periodontal parameters. Hierarchical linear regression analysis was used to explore the associations of PSS and general self-efficacy with post-treatment OHRQoL after adjusting for some demographic and periodontal variables. Asymptotic and resampling strategies were performed to explore the mediating role of general self-efficacy.

**Results:**

Initial periodontal therapy resulted in a significant improvement in the mean total score and all domains of OHRQoL and all periodontal parameters measured. In hierarchical linear regression analysis, clinical attachment loss (CAL) was significantly and positively associated with post-treatment OHRQoL score (β = 0.265, *p* < 0.01), while PSS (β = −0.303, *p* < 0.01) and general self-efficacy (β = −0.221, *p* < 0.01) were significantly and negatively associated with post-treatment OHRQL score, respectively. A significant mediating role of general self-efficacy (a*b = −0.139, BCa 95 % CI: −0.298, −0.011) in the association between PSS and post-treatment OHRQoL was revealed, and the proportion of the mediating role of general self-efficacy was 31.4 %.

**Conclusions:**

Initial periodontal therapy could significantly improve all aspects of OHRQoL. PSS and general self-efficacy could be the positive resources for improving OHRQoL after initial periodontal therapy among patients with periodontitis. General self-efficacy partly mediated the association between PSS and post-treatment OHRQoL.

## Background

Periodontitis is a chronic inflammatory disease, characterized by the progressive loss of the tooth-supporting supporting tissues, which include gingiva, periodontal ligament, cementum, and alveolar bone [1]. Periodontitis may not only cause oral discomforts, tooth worse or loss, but can also affect systemic health by increasing the patients’ risk for some serious health problems [2, 3]. In China, 85.5 % of adults over the age of 35 years are suffering from periodontitis [4]. The Chinese population posses more severe periodontitis popularity compared with people in most developed countries, as a result of the loss of self-consciousness about oral health [5, 6]. Of course, oral health-related problems in patients with periodontitis negatively affect their perceptions of oral health-related quality of life (OHRQoL) [7–10].

In addition to improvement in traditional objective endpoints, such as gingival inflammation and gains in attachment [11], subjective OHRQoL should be considered as an especially important endpoint to fully assess the efficacy of periodontal therapy for oral health [12, 13]. OHRQoL is a comprehensive outcome of the perception about physiological, psychological and social impacts of oral health problems. OHRQoL not only can reflect the impact of stomatognathic system, but also describes patients’ subjective experience of oral health and delivers information to complement objective clinical parameters. Because the duration of periodontal therapy is often lengthy, and its procedures could inflict some discomforts, periodontal therapy may not be truly effective when the assessment endpoint is patient’s perception [13]. Although periodontal therapy has a positive role on the OHRQoL of patients [14], it is also vital to find some positive resources from social and psychological aspects to promote the overall OHRQoL of patients after initial periodontal therapy.

Social support refers to the perception and actuality that individuals are cared for and have assistance available in times of need from other people in their supportive social networks [15]. Previous studies have shown that social support could contribute to OHRQoL among patients with different health problems [16–19]. Obviously, adequate and lasting social support will help patients overcome the discomforts during or after the periodontal treatment period. But, little is known about the relationship between OHRQoL and social support among patients with periodontitis after initial periodontal therapy.

Currently, some internally psychological constructs have been given increasing attention in research on the potential resources of OHRQoL with the rise of positive psychology [17, 20, 21]. General self-efficacy refers to a relatively stable belief in personal competence to deal with various stressful situations effectively [22]. Those who have greater confidence in their competence to execute decision tend to be more likely to attain desired goals, such as well-being and quality of life. Self-perceived and parent-reported general self-efficacy was important for the quality of life of adolescents with chronic conditions [23]. Reasonably, those with a high sense of oral health-related self-efficacy might have a high motivation for oral health-promoting behaviors, which would promote the maintenance of good oral health. However, very limited research work has been done to examine the positive effect of general self-efficacy on OHRQoL to our best knowledge.

In addition, the effect of external factors is often mediated by subjective evaluation and coping process that can be affected by personal self-efficacy beliefs for a stressful event. Previous studies have found that self-efficacy is a mediator in the association between social support and improved quality of life [24, 25]. Therefore, we hypothesized that social support predicts OHRQoL among patients with periodontitis after initial periodontal therapy, and the effect of social support is mediated by general self-efficacy.

With the purpose of improving the overall OHRQoL after initial periodontal therapy, we firstly assessed the effect of initial periodontal therapy on the OHRQoL of patients with periodontitis. Secondly, the associations of perceived social support (PSS) and general self-efficacy with post-treatment OHRQoL were examined. Thirdly, we explored the mediating role of general self-efficacy in the association between PSS and post-treatment OHRQoL.

## Methods

### Study sample

Firstly, we calculated the minimum sample size needed to assess the study’s hypothesis before sample recruitment by the formula: *n* = [(Z_α/2_ + Z_β_)S/δ]^2^. In the formula, α = 0.05, Z_α/2_ = 1.960; β = 0.10, Z_β_ = 1.282. S is the standard deviation of the difference of OHRQoL at baseline and post-treatment, which is estimated according to the results of previous studies and trial test. In this study, S = 3.0. δ is tolerable error, and δ = 0.3S = 0.9. Thus, *n* = 116.8. As a result, we identified 117 as the minimum sample size for this study.

From July 2014 to April 2015, a prospective case series study was conducted among consecutive outpatients with chronic periodontitis from the Department of Periodontics and Oral Biology at Stomatology Hospital of China Medical University. All eligible patients were invited to participate in the study by their treating or attending dentists in charge. Potential subjects were required to meet the following inclusion criteria: (1) were at least 18 years old; (2) were diagnosed with chronic periodontitis that included a minimum of four sites with probing depth (PD) ≥ 4 mm in different quadrants with radiographic evidence of bone loss and the presence of ≥16 teeth with a minimum of four molars; (3) had no extensive periodontal therapy in the past 6 months, (4) had clear consciousness and cognition. Patients were excluded if they had active caries, aggressive periodontitis or other oral diseases.

The Committee on Human Experimentation of Stomatology Hospital of China Medical University reviewed and provided the ethics approval for this study, and the study procedures were in accordance with the ethical standards. Participation was voluntary and anonymous, and participants were informed about the purpose and protocol of the study.

#### Procedure

After obtaining written informed consent, a total of 145 eligible patients responded to an OHRQoL questionnaire at baseline before scheduled initial periodontal therapy. Also, a periodontal examination was carried out at baseline. Firstly, the number of teeth was determined. In this study, all periodontal parameters were measured at four sites per tooth for full mouth. PD and gingival recession (REC) were measured using a Florida probe with a force of 0.25 N performed by the treating or attending dentists in charge. Clinical attachment loss (CAL) was the algebraic sum of PD and REC. For the three parameters, their values were rounded up to the nearest millimeter. Full-mouth bleeding scores (FMBS) were assessed to record the presence or absence of bleeding after PD was measured. Impacted teeth, grossly broken down teeth, inaccessible teeth and retained roots were excluded from the examination. The O’Leary plaque control record (PCR) was adopted to record the presence or absence of supragingival dental plaque. In addition, because CAL presents a good overall assessment of the severity of periodontal damage, our subjects were divided into 3 groups (initial, moderate and severe periodontitis) based on their CAL measurements [26].

The initial periodontal therapy mainly included scaling and root planning with local anesthesia, as well as standard oral hygiene instruction. A tooth with a hopeless prognosis was extracted. The scaling and root planning were performed using Gracey curets and an ultrasonic scaler. Oral hygiene was checked, and oral health instructions were given at each appointment.

After about 4 to 5 weeks for tissue healing, the second measurement was performed. Among the patients who completed measurement at baseline, 25 patients did not return within the prescribed time. There were 120 patients completed the second OHRQoL measurement and periodontal examination, along with PSS and general self-efficacy measurement, and they were subjected to data analysis.

All the questionnaires were completed in a quiet room. In the process of completing questionnaires, there was no interference caused by treating or attending dentists in charge as investigators, and explanation for any unclear item was provided to avoid any error and ensure data quality.

### Measures

#### Measurement of OHRQoL

OHRQoL was measured using the Chinese version of the short form of the Oral Health Impact Profile (OHIP-14) [27]. The OHIP-14 consists of 7 domains of oral health impact, including functional limitation, physical pain, psychological discomfort, physical disability, psychological disability, social disability and handicap. Each item is scored on a 5-point Likert scale ranging from 0 “never” to 4 “very often”. The additive scores were calculated by summing scores for total OHRQoL (ranging from 0 to 56) and each domain (ranging from 0 to 8), respectively. A higher score indicates that one experiences more negative impacts and a poorer OHRQoL. The Chinese OHIP-14 has been widely used among Chinese patients with oral diseases, and has adequate reliability and validity [28, 29]. In the present study, the Cronbach’s α coefficient of the OHIP-14 at baseline and the second measurement was 0.94 and 0.92, respectively.

#### Measurement of PSS

PSS was measured by the Chinese version of the Multidimensional Scale of Perceived Social Support (MSPSS) [30]. The MSPSS consists of 12 items, and measures perceived support from three social relationships: family, friends and significant others. Each item is scored on a 7-point Likert scale ranging from 1 “very strongly disagree” to 7 “very strongly agree”. The summed score of the MSPSS ranges from 12 to 84, and higher score indicates greater satisfaction with social support. The Chinese MSPSS has both good reliability and validity among various Chinese patients [31, 32]. The Cronbach’s α coefficient of the MSPSS was 0.97 in this study.

#### Measurement of general self-efficacy

General self-efficacy was measured by the Chinese version of the General Self-efficacy Scale (GSES) [33]. The GSES consists of 10 items that are scored on a 4-point Likert scale, ranging from 1 “not at all true” to 4 “exactly true”. The total score ranges from 10 to 40, and higher score indicates higher level of general self-efficacy. The Chinese GSES has also been adapted to Chinese patients with good reliability and validity [34, 35]. The Cronbach’s α value for the GSES was 0.93 in this study.

#### Demographic characteristics

Patients’ demographic characteristics including gender, age, marital status, educational level, average monthly income, payment type, family history, other chronic comorbidity and current smoking were collected. Age was collected as a continuous variable, and was divided into five groups: < 30, 30–39, 40–49, 50–59 and ≥60 years. Marital status was categorized as single/divorced/widowed/separated and married/cohabiting. Educational level was categorized as senior high school or below and junior college or above. Average monthly income (RMB) was categorized as ≤3000 and >3000 yuan. Payment type was categorized as medical insurance and self-payment. Family history was recorded as “yes” if a participant had a family history of periodontal disease. Other chronic comorbidity was defined as “yes” if a participant had ever been diagnosed with any common chronic disease, such as hypertension, diabetes and hepatitis). Two questions were adopted to assess current smoking status: (1) in your lifetime, have you smoked more than 100 cigarettes? (2) in the past 30 days, have you smoked a cigarette, even a puff? Participants were defined as current smokers if they answered “yes” to both questions.

### Statistical analysis

The demographic and periodontal variables were described with number (n), percentage (%), mean, standard deviation (SD) and median appropriately. The Wilcoxon matched-pairs signed-rank test was used to determine the difference between baseline and post-treatment OHRQoL scores and periodontal parameters. The Mann–Whitney U and Kruskal-Wallis tests were used to analyze the group differences of post-treatment OHRQoL scores. Correlations of periodontal parameters, PSS and general self-efficacy with post-treatment OHRQoL score were computed using the Spearman rank correlation. Hierarchical linear regression analysis was used to explore the associations of the groups of demographic, periodontal and psychosocial (PSS and general self-efficacy) variables with post-treatment OHRQoL. In Block 1, demographic variables were added. Periodontal variables were added in Block 2. PSS was added in Block 3, and general self-efficacy was added in Block 4. The values of F, R^2^, R^2^-changes (ΔR^2^), standardized regression coefficient (β) in each step of regression model were reported. Moreover, tolerance and variance inflation factor (VIF) were calculated to identify multi-collinearity. When general self-efficacy was added in regression model, a reduction in the size of direct regression coefficient of PSS on post-treatment OHRQoL or a disappearance of statistical significance indicated a potential mediation. Then, asymptotic and resampling (bootstrapping) strategies were used to examine the mediating role (a*b product) of general self-efficacy [36]. Based on 5000 bootstrap samples, the bias-corrected and accelerated 95 % confidence interval (BCa 95 % CI) of the a*b product was estimated, and a BCa 95 % CI excluding 0 indicated a significant mediating role. All analyses were conducted using SPSS for Windows, Ver. 13.0, and two-tailed *p* < 0.05 was considered to be statistically significant.

## Results

### Participant characteristics

Participant characteristics are presented in Table 1. Among the 145 eligible patients, there were 25 lost participants, and 120 patients completed OHRQoL measurement and periodontal examination both at baseline and the second assessment. The effective response rate was 82.8 % in this study. Among those subjects, 64.2 % (77) of patients were women. The participants were in the age range of 19–74 years (Mean ± SD: 43.82 ± 12.72). Of these patients, 78.3 % (94) were married/cohabiting, and 74.2 % (89) had a junior college or above education. Seventy-two participants (60.0 %) had an average monthly income level of ≤3000 yuan RMB, and 63 (52.2 %) had to pay for medical care themselves. In relation to the other health-related variables, 74 (61.7 %) participants reported a family history of periodontal disease, 38 (31.7 %) reported that they had at least one other chronic condition, 20 (16.7 %) were current smokers, and 51 (42.5 %) were severe periodontitis.

**Table 1:** Participant characteristics

Characteristic variables	*n* (%)
Gender	
Men	43 (35.8 %)
Women	77 (64.2 %)
Age (years)	
< 30	16 (13.3 %)
30–39	31 (25.8 %)
40–49	34 (28.3 %)
50–59	23 (19.2 %)
≥ 60	16 (13.3 %)
Mean (SD)	43.82 (12.72)
Median (Range)	43 (19–74)
Marital status	
Single/divorced/widowed/separated	26 (21.7 %)
Married/cohabiting	94 (78.3 %)
Educational level	
Senior high school or under	31 (25.8 %)
Junior college or above	89 (74.2 %)
Average monthly income (RMB, yuan)	
≤ 3000	48 (40.0 %)
> 3000	72 (60.0 %)
Payment type	
Medical insurance	57 (47.5 %)
Self-payment	63 (52.2 %)
Family history	
Yes	74 (61.7 %)
No	46 (38.3 %)
Other chronic comorbidity	
Yes	38 (31.7 %)
No	82 (68.3 %)
Current smoking	
Yes	20 (16.7 %)
No	100 (83.3 %)
Severity	
Initial	35 (29.2 %)
Moderate	34 (28.3 %)
Severe	51 (42.5 %)

*SD* standard deviation

### Effects of initial periodontal therapy on OHRQoL scores and periodontal parameters

OHRQoL scores and periodontal parameters at baseline and post-treatment are presented in Table 2. The mean total score and all domain scores of post-treatment OHRQoL showed significant decrease compared with those baseline scores. The initial periodontal therapy resulted in significant improvement in all periodontal parameters measured, with minimal tooth loss (mean number of lost teeth: 0.57).

**Table 2:** OHRQoL scores and periodontal parameters at baseline and post-treatment

Variables	Baseline	Post-treatment
OHRQoL scores		
Total score	16.67 (12.10)	7.05 (7.16) **
Functional limitation	1.83 (2.21)	1.08 (1.48) **
Physical pain	3.55 (1.81)	1.52 (1.53) **
Psychological discomfort	2.59 (2.26)	0.91 (1.33) **
Physical disability	2.68 (2.09)	1.25 (1.49) **
Psychological disability	2.43 (2.01)	0.99 (1.27) **
Social disability	1.67 (1.93)	0.61 (1.10) **
Handicap	1.93 (1.96)	0.69 (1.14) **
Periodontal parameters		
No. of teeth (*n*)	26.96 (2.24)	26.39 (2.71) **
PD (mm)	3.00 (0.75)	2.53 (0.65) **
CAL (mm)	2.66 (1.46)	2.50 (1.47) *
FMBS (%)	35.07 (23.52)	19.99 (16.13) **
PD ≥ 4 mm (%)	25.83 (19.34)	12.93 (12.74) **
PCR (%)	56.66 (20.75)	37.57 (15.51) **

*OHRQoL* oral health-related quality of life, *PD* probing depth, *CAL* Clinical attachment loss, *FMBS* full-mouth bleeding scores, *PCR* plaque control record

*p < 0.05, **p < 0.01

### Univariate analyses results predicting post-treatment OHRQoL

Group differences of post-treatment OHRQoL scores and correlations of periodontal parameters, PSS and general self-efficacy with post-treatment OHRQoL score are presented in Table 3. The statistically significant relations between post-treatment OHRQoL score and demographic variables were not found in this study. No. of teeth, PSS and general self-efficacy had negative correlations with post-treatment OHRQoL score, whereas PD and PD ≥ 4 mm (%) were positively correlated with post-treatment OHRQoL score.

**Table 3:** Univariate analyses results of the variables in relation to post-treatment OHRQoL score

Variables	OHRQoL score	*p*
Gender		0.360
Men	6.23 (6.86)	
Women	7.51 (7.33)	
Age (years)		0.151
< 30	6.88 (7.92)	
30–39	4.45 (4.29)	
40–49	9.41 (8.49)	
50–59	7.70 (6.37)	
≥ 60	6.31 (7.84)	
Marital Status		0.299
Single/divorced/widowed/separated	8.96 (8.78)	
Married/cohabiting	6.52 (6.60)	
Educational level		0.895
Senior high school or under	6.42 (5.94)	
Junior college or above	7.27 (7.56)	
Average monthly income (RMB, yuan)		0.966
≤ 3000	7.46 (7.96)	
> 3000	6.78 (6.62)	
Payment type		0.449
Medical insurance	6.09 (5.95)	
Self-payment	7.92 (8.05)	
Family history		0.639
Yes	6.54 (6.70)	
No	7.87 (7.85)	
Other chronic comorbidity		0.152
Yes	8.37 (7.60)	
No	6.44 (6.91)	
Current smoking		0.713
Yes	7.05 (6.36)	
No	7.05 (7.34)	
Spearman r with OHRQoL score		
Age (years)	0.077	0.432
No. of teeth (n)	−0.187	0.039
PD (mm)	0.215	0.019
CAL (mm)	0.347	<0.001
FMBS (%)	0.164	0.077
PD ≥ 4 mm (%)	0.263	0.004
PCR (%)	0.059	0.508
PSS	−0.504	<0.001
General self-efficacy	−0.492	<0.001

*OHRQoL* oral health-related quality of life, *PD* probing depth, *CAL* Clinical attachment loss, *FMBS* full-mouth bleeding scores, *PCR* plaque control record, *PSS* perceived social support

### Hierarchical linear regression results predicting post-treatment OHRQoL

Hierarchical linear regression analysis results are presented in Table 4. In periodontal parameters, only CAL was significantly and positively associated with post-treatment OHRQoL score (β = 0.265, *p* < 0.01) in the final model. PSS was significantly and negatively associated with post-treatment OHRQoL score (β = −0.443, *p* < 0.01), and it accounted for an additional 15.8 % variance to the prediction of post-treatment OHRQoL in Block 3. General self-efficacy represented significant and negative association with post-treatment OHRQoL (β = −0.221, *p* < 0.01), and accounted for an additional 2.5 % variance in Block 4. In addition, the effect of PSS on post-treatment OHRQoL in Block 4 (β = −0.303, *p* < 0.01) was reduced compared with that in Block 3, as indicated by a smaller β absolute value.

**Table 4:** Hierarchical linear regression analysis results of the variables in relation to post-treatment OHRQoL score

Variables	Block 1 (β)	Block 2 (β)	Block 3 (β)	Block 4 (β)
Gender	0.113	0.163	0.130	0.083
Age	0.176	0.024	−0.078	−0.064
Marital status	−0.184	−0.194	−0.132	−0.113
Educational level	0.156	0.055	0.079	0.082
Average monthly income	−0.026	0.098	0.088	0.074
Payment type	0.173	0.137	0.092	0.091
Family history	0.121	0.140	0.120	0.137
Other chronic comorbidity	−0.084	−0.067	−0.087	−0.074
Currently smoking	−0.015	−0.022	−0.055	−0.069
No. of teeth (n)		−0.125	−0.085	−0.057
PD (mm)		−0.023	−0.082	−0.030
CAL (mm)		0.298 **	0.273 **	0.265 **
FMBS (%)		−0.209	−0.177	−0.150
PD ≥ 4 mm (%)		0.332 *	0.240	0.206
PCR (%)		−0.094	−0.028	−0.030
PSS			−0.443 **	−0.303 **
General self-efficacy				−0.221 *
F	1.272	2.627 **	4.899 **	5.046 **
R^2^	0.096	0.279	0.437	0.462
ΔR^2^	0.096	0.183 **	0.158 **	0.025 *

*OHRQoL* oral health-related quality of life, *PD* probing depth, *CAL* Clinical attachment loss, *FMBS* full-mouth bleeding scores, *PCR* plaque control record, *PSS* perceived social support. Gender: women vs. men; Marital status: married/cohabitation vs. single/divorced/widowed/separated; Educational level: junior college or above vs. senior high school or under; Average monthly income: > 3000 yuan vs. ≤ 3000 yuan; Payment type: self-payment vs. medical insurance; Family history: no vs. yes; Other chronic comorbidity: no vs. yes, Currently smoking: yes vs. no

*p < 0.05, **p < 0.01

### Mediating role of general self-efficacy

PSS was significantly and positively associated with general self-efficacy (*a* = 0.615, *p* < 0.01). General self-efficacy was significantly and negatively associated with post-treatment OHRQoL score after controlling for demographic and periodontal variables and PSS (*b* = −0.227, *p* < 0.01). Thus, there was a significant mediating role of general self-efficacy (a*b = −0.139, BCa 95 % CI: −0.298, −0.011) in the association between PSS and post-treatment OHRQoL. The proportion of total effect of PSS on post-treatment OHRQoL by mediation was calculated with the formula: (a*b)/total effect. The proportion of the mediating role of general self-efficacy was 31.4 %.

## Discussion

The present study indicated that initial periodontal therapy could significantly improve all aspects of OHRQoL and the objective endpoints of periodontitis. On the basis of positive changes, PSS directly and indirectly through promoting general self-efficacy further made new contributions to the improvement of OHRQoL after initial periodontal therapy, and they could be effectively external and internal resources.

Among clinically periodontal parameters, only CAL showed a positively indicative effect on post-treatment OHRQoL score. CAL is an objective indicator that represents the degree of periodontal support tissue destruction, and it is also an important sign of the difference between gingivitis and periodontitis. The degree of CAL can be described through a combination of epithelium to glaze tooth coronal bone distance [37]. Although an initial periodontal therapy has been performed, patients who had a higher CAL would report worse OHRQoL yet [38, 39]. One possible reason is that patients with higher loss attachment measurement by CAL could have more mobility and function limitations which were perceived. The result indicated that the degree of CAL could be used as an objectively clinical indicator of post-treatment OHRQoL. However, the other demographic and periodontal variables collected in this study were not associated with post-treatment OHRQoL. Saito et al. reported that no association was observed between age, gander, PD, CAL, FMBS and PD ≥ 4 mm and post-treatment OHRQoL [13]. Also, the result from a prospective cohort study indicated that age, gender and tobacco habits of the participants did not have a significant impact on OHRQoL after treatment [40]. One possible reason could be that periodontal therapy is the most important factor of post-treatment OHRQoL, which may weaken the effects of other factors.

The positive effects of social support on health outcomes and quality of life have been widely confirmed. Supportive social network could help individuals cope with various stressful events and serve as a buffer against their negative health impacts through behavioral, psychological and physiological pathways. Brennan and Spencer found that high social support was associated with fewer decayed teeth and less negative impact on OHRQoL compared to low support in univariate analyses, and social support had negative associations with missing teeth and caries experience after adjusting for demographic factors and oral health-related behaviors among young adults [17]. Gabardo et al. revealed that those with less social support presented higher odds of reporting worse self-perception of oral health in Southern Brazilian adults [16]. Lamarca et al. found that individual social capital level (social support and social networks) were independently associated with high OHRQoL in pregnant women. The study findings suggested that the quality of personal and social resources could be more important for OHRQoL than the neighbourhoods where the women live [18]. Consistent with these findings, the post-treatment OHRQoL of patients with periodontitis could be improved by increased PSS level in our study.

In the present study, general self-efficacy played a positive role in improving OHRQoL after initial periodontal therapy. This finding was in accordance with results from some previous studies. Broder et al. reported that self-efficacy were positively associated with OHRQoL among youths with cleft [20]. In older people, Weening-Verbree et al. found that self-efficacy was one of the determinants that are often addressed in implementation strategies for successful improvement of oral health care [21]. In symptom management, patients with high level of self-efficacy had less psychological distress, better adjustment and quality of life compared to patients with low level of self-efficacy [41]. Moreover, those with high self-efficacy often show high adherence and satisfaction to their treatment received. In addition, general self-efficacy partially mediated the association between PSS and post-treatment OHRQoL in this study. Thus, PSS not only showed direct effect on OHRQoL among patients with periodontitis after initial periodontal therapy, but also had indirect effect by promoting general self-efficacy.

These findings lead us to believe that both social support and general self-efficacy are important to successfully improve patients’ OHRQoL after initial periodontal therapy. In addition to encouraging increase the actual support from various social relationships of patients, the perception of social support could be enhanced through specific intervention based on cognitive-behavioral therapy. Nurse-dominated intervention had resulted in higher quality of life or psychological well-being as a result of improved self-efficacy among different patient groups [41, 42]. The intervention mainly included mastery experiences, vicarious experiences and verbal persuasion. Schwarzer et al. reported that university students in intervention group that received a brief self-regulatory treatment received specific gains, which were documented for self-efficacy and self-monitoring. As a result, the intervention finally led to an increase in dental flossing in this population [43]. Patients with neuroendocrine tumors could improve their ability to cope with disease, problem-solve and physical status, and reduce stress following an educational intervention based on the principles of self-efficacy [44]. However, intervention targeted toward enhancing PSS and general self-efficacy of patients with periodontitis should be developed to improve their post-treatment OHRQoL.

There are several limitations to the present study. First, it was conducted at a single regional treatment center, and no differences in demographic characteristics and clinical factors were detected between participants and those who refused participation. The second limitation was the absence of control group. As a result, the interpretation of the results should be made with caution. Nevertheless, the Department of Periodontics and Oral Biology at Stomatology Hospital of China Medical University is an important service center for treating periodontal diseases in northeastern China. There was a high effective response rate (82.8 %). Moreover, the primary purpose of this study was to explore the associations of PSS and general self-efficacy with post-treatment OHRQoL among patients with periodontitis. Thus, these facts have guaranteed the generalizability of our findings to some extent. However, further multi-center and large sample size research with a control group could provide a good representation of patients with periodontitis and contribute to the generalization of our findings. Third, study variables were mainly detected using self-report measures. There was a possibility of recall and reporting bias, and associations among these variables might be influenced. Some effective process control measures were carried out to minimize the potential common-method bias. Fourth, further studies are needed to examine the effects of other positive psychological constructs on OHRQoL and the integrating effect.

## Conclusions

Initial periodontal therapy could significantly improve all aspects of OHRQoL. PSS and general self-efficacy could be positive resources for improving OHRQoL after initial periodontal therapy among patients with periodontitis. General self-efficacy partly mediated the association between PSS and post-treatment OHRQoL. PSS not only could directly improve patients’ OHRQoL after initial periodontal therapy, but also have an indirect effect on post-treatment OHRQoL by promoting general self-efficacy.

## Abbreviations

BCa 95 % CI, bias-corrected and accelerated 95 % confidence interval; CAL, clinical attachment loss; FMBS, full-mouth bleeding scores; GSES, General Self-efficacy Scale; MSPSS, Multidimensional Scale of Perceived Social Support; OHIP, Oral Health Impact Profile; OHRQoL, oral health-related quality of life; PCR, plaque control record; PD, probing depth; PSS, perceived social support; REC, gingival recession; SD, standard deviation; VIF, variance inflation factor.
